# Development and Validity of the Nurse Presenteeism Questionnaire

**DOI:** 10.3389/fpsyg.2021.679801

**Published:** 2021-05-21

**Authors:** Geyan Shan, Shengnan Wang, Kai Feng, Wei Wang, Shujie Guo, Yongxin Li

**Affiliations:** ^1^Institute of Psychology and Behaviour, Henan University, Kaifeng, China; ^2^North China University of Water Resources and Electric Power, Zhengzhou, China; ^3^Nursing Department, Henan Province People’s Hospital, Zhengzhou, China

**Keywords:** presenteeism, nurse presenteeism questionnaire, reliability, validity, China

## Abstract

This study aimed to develop and test the reliability and validity of a multi-item nurses’ presenteeism behaviour questionnaire. Study 1 administered the Nurse Presenteeism Questionnaire (NPQ) to 250 Chinese nurses. Study 2, surveyed 650 nurses with the NPQ, the Sickness Presenteeism Questionnaire, the Stanford Presenteeism Scale, the General Health Questionnaire, and the Emotional Exhaustion Scale using convenience sampling. After item analysis, the subjects were randomly divided into two groups to verify the questionnaire structure. Study 1 revealed the nurses’ core symptoms when they go to work with illness, and the NPQ with 11 items was developed. Study 2’s item analysis revealed that 11 NPQ items had good discrimination (*t* = 22.67∼36.11, *p* < 0.01) and high homogeneity. Besides, the scale had good reliability (Cronbach’s = 0.93) and external criterion validity (*r* = 0.24∼0.84, *p* < 0.01). Thus, the NPQ can be used to measure presenteeism behaviour in nursing.

## Introduction

In an Eastern cultural context, persisting with work in spite of illness has been regarded as a sign of diligence and dedication since antiquity, and such behaviours have been reported by mainstream media as meritorious deeds in China for the past several decades. However, in recent years, the widespread occurrence of negative social phenomena, such as “overwork death,” has not only attracted people’s attention to health, but also started a debate on working despite illness, which has become a hot topic in the society. Besides, this phenomenon has received close attention from scholars (e.g., Aronsson et al., 2000; Johns, 2010; Lu et al., 2013b; Sun and Zhang, 2015; Yang et al., 2019). They have defined this behaviour as “presenteeism behaviour,” in which an individual, who is supposed to be on leave for being sick, still attends work in poor health (Aronsson et al., 2000). Studies have shown that nurses are one of the professional groups with high and frequent presenteeism behaviour (Pilette, 2005; Bergström et al., 2009). Freeling et al. (2020) summarised studies on nurse presenteeism behaviour in multiple countries from 2006 to 2018, revealing that the incidence of nurse presenteeism behaviour ranged from 15.74% (*N* = 147) (Brborovic et al., 2016) to 86.96% (*N* = 951) (Dellve et al., 2011). In most studies, the average incidence of the behaviour was approximately at 50%–sometimes, even higher. In China, Shan et al. (2021) discovered that 94.25% of Chinese nurses reported that they had engaged in presenteeism behaviour in the preceding 6 months, and the incidence was 82.08% in their direct leaders’ eyes. Thus, it is urgent to attach importance to the presenteeism behaviour in Chinese nursing professions.

In 2016, the Central Committee of the Communist Party of China and The State Council issued *The Program of Healthy China 2030* (hereinafter referred to as *the program*), which elevates people’s health issues as the fundamental strategy for China’s development. *The program* puts forward new requirements for national health and new targets for optimising health services. It clearly points out that the medical service system should be further improved to facilitate the quality of medical services (The State Council of the CPC Central Committee, 2016). As promoters of human health, guardians of healthy life, and communicators of health literacy, nurses make up the highest proportion of China’s medical service teams and shoulder the important task of people’s health development.

Moreover, the World Innovation Summit of Health (WISH) suggested that nurses and midwives are the most critical players in universal healthcare (Crisp et al., 2018). The improvement of the national health level is closely related to the quality of nurse care. However, the presenteeism behaviour of nurses tends to affect the treatment and rehabilitation of their patients, reduce their nursing quality, and introduce negative effects, such as an increase in the number of falls of patients and drug errors (Letvak et al., 2012). Nurse presenteeism behaviours will not only affect nurses’ physical and mental health, reduce job satisfaction and engagement, and increase job burnout, but also bring financial burden and productivity loss to medical organisations (Demerouti et al., 2009; Letvak et al., 2012; Kandemir Türe and Bayram, 2017; Zhang et al., 2018; Shan et al., 2021).

To sum up, the common occurrence of unhealthy work behaviours (i.e., presenteeism behaviours) among nurses is not only related to their own health, but also has a profound impact on the promotion of public health and the improvement of the country’s overall medical service and quality. This further illustrates the significance of investigating nurses’ presenteeism behaviour.

Although presenteeism behaviour has caused widespread concern among scholars in areas such as occupational health psychology, organisational behaviour, and human resource management, research on presenteeism has been mainly conducted in Europe and the United States, while it is still in its infancy in China. Furthermore, research on presenteeism behaviour against the background of Chinese culture is scarce, and the corresponding measurement tools need to be further improved (Johns, 2010; Sun and Zhang, 2015; Zhang and Li, 2016; Li et al., 2019). Generally, scholars have different interests in measuring presenteeism behaviour, and their main measurement methods differ. They can be divided into two categories: one that focuses on the measurement of productivity loss caused by the behaviour, and the other that focuses on the behaviour itself. The former defines presenteeism behaviour based on its negative outcome (Johns, 2010). Moreover, the ambiguous nature of productivity in many jobs and the implicit characteristics of the link between health and productivity may make it difficult to accurately estimate presenteeism related productivity losses (Johns, 2012). Therefore, at present, most scholars tend to interpret presenteeism behaviour as the behaviour of working in an unhealthy state” based on the nature of presenteeism behaviour, instead of making positive or negative judgments on the behaviour itself (Li et al., 2019; Ruhle et al., 2019).

An effective measurement is the basic condition for further research. Presently, the measurement of presenteeism behaviour is mainly based on unverified single- or double-item measurements (Miraglia and Johns, 2016; Lohaus and Habermann, 2019). To measure presenteeism behaviour, Aronsson et al. (2000) posed this question: “Has it happened over the previous 12 months that you have gone to work despite feeling that you really should have taken sick leave due to your state of health?” Participants were asked to report the frequency of the behaviour based on a four-point rating method. Such questionnaires limit the health status of individuals at the time of the occurrence of presenteeism to a “health status that warrants sick leave.” Demerouti et al. (2009) simplified Aronsson et al.’s (2000) measurement by posing the question to measure presenteeism behaviour: “Has it happened over the previous 12 months that you have gone to work despite feeling sick?” Participants were asked to answer with “yes/no” to measure their presenteeism behaviour although their health condition was not defined. Subsequently, Lu et al. (2013a) used a two-item questionnaire to measure the frequency of presenteeism behaviour. The two items were: “Although you feel sick, you still force yourself to go to work” and “Although you have physical symptoms such as headache or backache, you still force yourself to go to work.” Such a measure exceeds the limit of individuals’ health conditions, but the term “force” implies that the individuals who engaged in presenteeism behaviour may have required extra effort to finish their work.

Therefore, due to the lack of a unified development standard, the emphasis of measurement tools developed by various scholars also differs. Moreover, different scholars used different ways of expression, answers, and recall cycles to measure the content of presenteeism behaviour, which also restricted the comparison of the measured results of presenteeism behaviour (Skagen and Collins, 2016; Hou, 2019; Ruhle et al., 2019). In addition, studies have shown that participants’ decision to be absent when they are ill is closely related to the symptoms and severity of the disease from which they suffer (Kaldjian et al., 2019). Thus, this study targets nurses as the research subjects in developing a multi-item presenteeism behaviour questionnaire, focusing on nurse characteristics by considering the role of disease symptoms and severity on the decision to engage in presenteeism; it then tests the reliability and validity of the questionnaire.

In this study, we intend to compile the disease symptoms despite which nurses go to work with illness through an open-answered questionnaire survey. Considering that individuals have different sensitivities to symptoms (Nielsen et al., 2009) and that there are many types of diseases and symptoms, it is not appropriate to make a unified classification. We focused on whether presenteeism behaviour occurred in situations in which participants experienced a physical state of being able to choose between engaging in presenteeism and taking leave. Therefore, we excluded diseases or symptoms that are seriously severe (i.e., that involve lack of behavioural competence), and defined the severity of the disease as follows: although behavioural competence is basically normal, having a significant sense of discomfort, which can be overcome or mitigated with a certain amount of willingness and effort.

In order to examine the external validity of the Nurse Presenteeism Questionnaire (NPQ), the presenteeism-related variables were examined. First, although the number of questions in the Sickness Presenteeism Questionnaire (SPQ) by Lu et al. (2013b) was too small to conduct a test of validity, previous studies confirmed that it has good internal consistency reliability (Lu et al., 2014). Moreover, 90% of Chinese studies on presenteeism behaviour have applied this questionnaire. Thus, this study not only took the SPQ as the basic reference for the development of the NPQ, but also regarded it as an important criterion variable. The aim was to develop a questionnaire on nurses’ presenteeism behaviour so that scholars can flexibly choose the appropriate measurement tools according to their own research needs. Previous studies had confirmed that presenteeism behaviour is closely related to the individual’s health status (e.g., Skagen and Collins, 2016; Arjona-Fuentes et al., 2019), and health-related productivity losses (Rantanen and Tuominen, 2011; Li et al., 2019); moreover, individual emotional exhaustion is also closely related to presenteeism behaviour and its resulting productivity loss (Neto et al., 2017; Ferreira et al., 2019; Zhang et al., 2020). Therefore, this study examined individuals’ general health status, health-related productivity loss, and emotional exhaustion as the external criteria of the NPQ.

## Materials and Methods

### Study 1

The aim of Study 1 was to investigate the core symptoms of nurse presenteeism through an open-ended survey and generate the item pool, obtain the initial version of the NPQ.

#### Participants and Procedure

In this study, the open-ended questionnaire survey was conducted among a total of 250 nurses from five hospitals located in Henan province, China, through convenience sampling. After data cleaning, the questionnaires of 215 nurses were included for analysis with an effective response rate of 86.00%. In this survey, nurses were all females whose ages ranged from 20 to 44 years, with an average age of 29.18 years (SD = 4.47). Their nursing tenure ranged from 0.5 to 29 years, with an average of 7.11 years (SD = 4.69). The departments in which nurses worked included internal medicine, surgery, ophthalmology, paediatrics, obstetrics and gynaecology, emergency room, and outpatient service. Descriptive statistics was shown in Table 1, which included gender, marital status, and technical title.

**TABLE 1:** Descriptive statistics for all samples (*N*_1_ = 215, *N*_2_ = 572).

		Study 1 *N*_1_ = 215	Study 2 *N*_2_ = 572
Gender	Female	215 (100%)	559 (97.73%)
	Male	–	13 (2.27%)
Marital status	Married	149 (69.30%)	337 (65.03%)
	Unmarried	66 (30.70%)	200 (34.97%)
Technical title	Student nurse	4 (1.86%)	12 (2.10%)
	General nurse	42 (19.53%)	91 (15.91%)
	Senior nurse	111 (51.63%)	211 (36.89%)
	Professor of nursing	88 (26.98%)	252 (43.88%)
	Not respond	–	6 (1.05%)

The survey procedure was as follows: First, an open-ended question was posed to nurses to collect qualitative data about the various diseases and symptoms they experienced during presenteeism. Second, to determine the core diseases and symptoms of nurse presenteeism, the qualitative data were summarised and analysed repeatedly. Then, the item pool was constructed based on these core items to form the initial version of the NPQ. Two nursing professionals who were experienced in scientific research and clinical practice (both holding a master’s degree in nursing and having worked in clinical nursing for more than 10 years) and three experts in psychology who were long engaged in scientific research (one professor, one postdoctoral student, and one doctoral student) were invited to jointly evaluate the content validity of this questionnaire. The items were modified and improved according to the suggestions of experts. Finally, the preliminarily version of the NPQ was developed after the content and expression of these items were unanimously approved by the expert group.

#### Measures

Apart from general demographic characteristics such as gender, age, nursing tenure, marital status, technical title, and work units, the SPQ (Lu et al., 2013a) was adopted among nurses to collect data as the basic reference of this open-ended question survey. SPQ comprises two items, namely: “Although you felt sick, you still forced yourself to go to work” and “Although you had physical symptoms such as a headache or backache, you still forced yourself to go to work.” Participants were required to rate how often they had experienced presenteeism during the previous 6 months. Each item was rated on a four-point scale (1 = never, 2 = once, 3 = 2–5 times, 4 = more than five times), with high scores representing more frequent instances of presenteeism. Then, the qualitative research data of core symptoms were collected using an open-ended questionnaire comprising one item, namely: “What kinds of diseases or symptoms (i.e., felling “sick” as mentioned above) did you have when you should have asked for sick leave but still turned up to work, in addition to the headache or back pain that is mentioned above? Please list them in the line below.”

#### Analysis and Results

From the responses to this open-ended question, a total of 499 original expressions of diseases and symptoms were obtained from 215 participants. Through the initial screening, the combined expression of symptoms was split. For example, “nausea and vomiting” was split into “nausea” and “vomiting” and “pain in waist and leg” was split into “waist pain” and “leg pain.” A total of 504 expressions of diseases and symptoms were thus obtained. Subsequently, through a further screening, some expressions that were beyond the scope of diseases or symptoms were deleted (e.g., being unwell, sick, unhappy, worried about my child, and having a scheduling conflict with parent–child activities), and a total of 487 expressions of diseases and symptoms were retained.

To facilitate further analysis of core symptoms, the unified expression of diseases and symptoms was coded. For example, “*teng*” and “*tong*” are different Chinese words, but they have the same meaning as both express an ache. Moreover, compared to “*teng*,” “*tong*” describes the physiological state of participants and the uncomfortable feeling caused by disease, which is more formal, so we uniformly coded all these feelings as “*tong*.” Similarly, other symptoms with the same meaning were coded with the same word. For instance, “loose bowels” and “diarrhoea” were coded as “diarrhoea,” “vertigo,” and “dizziness” were coded as “dizziness,” and “severe nasal congestion” was coded as “nasal congestion.”

Based on this preliminary collation, a total of 124 types of diseases and symptoms was gathered, including 24 diseases (such as scapulohumeral periarthritis, upper respiratory tract infection, ligament strain) from 33 responses, which accounted for 6.78% of the total responses, and 100 symptoms (such as fever, cold, and headache) from 454 responses, which accounted for 93.22% of the total responses. Then, these diseases and symptoms were further summarised; 30 categories of symptoms and 19 categories of diseases were obtained, as shown in Table 2 and Table 3.

**TABLE 2:** Preliminary summary of symptom categories.

	Symptom Category	Frequency	Subcategory of Symptoms (Frequency)
1	Fever	66	Fever (61), low-grade fever (3), high fever (2)
2	Dizziness	40	Dizziness (39), almost fainting (1)
3	Cold	39	Cold (36), bad cold (1), nasal obstruction (2)
4	Lumbago	38	Osphyalgia (33), waist (1), backache (2), lumbar pain (1), soreness of waist (1)
5	Abdominal pain or discomfort	29	Stomachache (14), bellyache (11), lower abdomen pain (1), abdominal discomfort (1), abdominal tenderness (1), rebounding pain (1)
6	Palpitation or being flustered	29	Being flustered (19), occasionally flustered (1), palpitation (7), severe palpitations (need to take medicine, 1), tachycardia (1)
7	Psychological discomfort	24	Anxiety (6), high psychological pressure (3), psychological discomfort (2), mental stress (2), be agitated (1), fretfulness (1), fear (1), in bad mood (1), emotion changes (1), psychological illness (1), mood annoyed (1), uncomfortable mood (1), high pressure (1), depressed (2)
8	Pain or discomfort during menstruation	23	Dysmenorrhea (9), menstrual pain (8), menstrual abdominal pain (1), menstrual bellyache (1), menstrual period pain (1), menstrual period stomach pain (1)
9	Stomachache or stomach discomfort	21	Stomachache (17), stomach discomfort (2), hunger-related stomach cramps (1), flatulence (1)
10	Headache	17	Headache (16), recurrent migraine (1)
11	Leg pain or swelling	17	Leg pain (16), sore and swollen leg (1)
12	General malaise	15	Sore and swollen (10), exhausted (1), whole body ache (1), panidrosis (1), malaise (1), discomfort from sitting or standing for too long (1)
13	Eye discomfort	11	Ophthalmodynia (7), asthenopia (1), dry eye (1), giddiness (1), blurring of vision (1)
14	Pain in the neck or cervical spine	10	cervical pain (6), neck pain (4)
15	Nausea	9	Nausea (9)
16	Diarrhoea	9	Diarrhoea (9)
17	Chest tightness or chest discomfort	9	Chest distress (7), discomfort of the precordial area (1), pain in the chest (1)
18	Cough	8	Cough (8)
19	Lack of sleep	7	Lack of sleep (5), insomnia (2)
20	Foot pain	6	Foot pain (3), pain in the joints of the feet (1), heel pain (1), feet osteoproliferation pain (1)
21	Emesis	5	Emesis (5)
22	Pregnant reaction	4	Pregnant reaction (2), pregnancy discomfort (1), abdominal pain during pregnancy (1)
23	Pain or swelling in the extremities	4	Arm pain (1), edema of lower extremity (1), lower limb acid bilges (1), ache of lower limb (1)
24	Joint discomfort	4	Joint muscle soreness (1), joint pain (1), knee joint pain (2)
25	Toothache	2	Toothache (2)
26	Respiratory disturbance	2	Respiratory disturbance (1), breathing hard (1)
27	Omodynia	2	Shoulder pain (1), scapulalgia (1)
28	Wrist discomfort	2	Numb hand (1), wrist pain (1)
29	Interpulmonary neuralgia	1	Interpulmonary neuralgia (1)
30	Nosebleed	1	Nosebleed (1)

**TABLE 3:** Preliminary summary of disease types.

	Symptoms	Frequency	Subcategory of Symptoms (Frequency)
1	Strain or sprain	6	Ligament injury (1), twisted foot (1), sprain (1), mild sprain of foot (1), foot sprain (1)
2	Glucopenia	5	Glucopenia (5)
3	Lumbar disc herniation	3	Lumbar disc herniation (3)
4	Periarthritis of shoulder	2	Periarthritis of shoulder (2)
5	Anaemia	2	Periarthritis of shoulder (2)
6	Lumbar muscle degeneration	2	Lumbar muscle degeneration (2)
7	Slipped disc	1	Slipped disc (1)
8	Ankle ligament injury	1	Ankle ligament injury (1)
9	Tenosynovitis	1	Tenosynovitis (1)
10	Varicosity	1	Varicosity (1)
11	Spasticity	1	Spasticity (1)
12	Kidney stone	1	Kidney stone (1)
13	Pre-excitation syndrome	1	Pre-excitation syndrome (1)
14	Upper respiratory infection	1	Upper respiratory infection (1)
15	Tonsillitis	1	Tonsillitis (1)
16	Cervical spondylosis	1	Cervical spondylosis (1)
17	Acute gastroenteritis	1	Sequela of foot injury (1)
18	Sequela of foot injury	1	Sequela of foot injury (1)
19	Gout	1	Gout (1)

There is great complexity and variation not only among the types of diseases afflicting the general population, but also with regard to the feelings they cause individuals; moreover, the expression of disease is also unique. In this study, the expression of disease accounts for less than 7% among nurses with high health literacy, which means that these expressions are uncommon with regard to the expressions of daily life. Therefore, we focus on the physical symptoms of presenteeism rather than the type of disease in this study. Analogously, mental and mood symptoms have been excluded from the questionnaire for two reasons. First, only a few participants (less than 5%) reported that they experienced mental illness (only one response) or mood-related symptoms during presenteeism in this study, which means that they were not widespread symptoms of presenteeism. And existing meta-analysis study also indicated that the overall relationship between mental health and presenteeism was negligible (Miraglia and Johns, 2016). Second, a few participants reported that the mood factor falls within the scope of being “sick,” but bad mood is hard to be the cogent reason to take sick leave in Chinese workplace culture. The evaluation of mood has strong subjectivity and instantaneity, which is easily affected by the environment. Accordingly, this study primary considered the objective aspects of physical symptoms as the conditions of presenteeism, disregarding the subjective aspects of mental state or mood.

In order to avoid the influence of stereotyped thinking, after an intentional interval of 1 month, we merged the symptoms of nurse presenteeism to obtain 18 types of symptoms (from 430 responses), as shown in Table 4. Based on the sorting of frequency, the top 10 core symptoms were selected as the main items of the NPQ, which accounted for 92.09% of the total responses. Moreover, to make the questionnaire as comprehensive as possible, another item was added to ensure that the other symptoms could be covered, namely, “Although you had other physical symptoms, you still persevered in going to work.”

**TABLE 4:** The core symptoms when presenteeism occurred and corresponding scale items.

	Symptoms	Frequency	Subcategory of Symptoms (Frequency)	Corresponding Item
1	Fever	66	Fever (61), low-grade fever (3), high fever (2)	Although you had a fever, you still persevered in going to work
2	Dizziness or Headache	57	Dizziness (39), almost fainting (1), headache (16), recurrent migraine (1)	Although you felt dizzy or had a headache, you still persevered in going to work
3	Abdominal pain or discomfort (include Pain or discomfort during menstruation)	52	Stomachache (14), bellyache (11), lower abdomen pain (1), abdominal discomfort (1), abdominal tenderness (1), rebounding pain (1), dysmenorrhea (9), menstrual pain (8), menstrual abdominal pain (1), menstrua (l), bellyache (1), menstrual period pain (1), menstrual period stomach pain (1)	Although you felt abdominal pain (including menstrual pain), you still persevered in going to work
4	Cold (include nasal obstruction and cough)	47	Cold (36), bad cold (1), nasal obstruction (2), cough (8)	Although you had a cold (e.g., stuffy nose or cough), you still persevered in going to work
5	Palpitation or being flustered chest tightness or chest discomfort respiratory disturbance	40	Being flustered (19), occasionally flustered (1), palpitation (7), severe palpitations (need to take medicine, 1), tachycardia (1), chest distress (7), discomfort of the precordial area (1), have pain in the chest (1), respiratory disturbance (1), breathing hard (1)	Although you felt chest distress, shortness of breath, or palpitations, you still persevered in going to work
6	Lumbago	38	Osphyalgia (33), waist (1), backache (2), lumbar pain (1), soreness of waist (1)	Although you felt discomfort in the lower back, you still persevered in going to work
7	Pain or swelling in the extremities	33	Leg pain (16), sore and swollen leg (1), arm pain (1), edema of lower extremity (1), lower limb acid bilges (1), +ache of lower limb (1), numb hands (1), wrist pain (1), Joint muscle soreness (1), joint pain (1), knee joint pain (2), foot pain (3), pain in the joints of the feet (1),heel pain (1), feet osteoproliferation pain (1), shoulder pain (1), scapulalgia (1)	Although you felt pain or swelling in limbs (and joints), you still persevered in going to work
8	Stomachache or discomfort	21	Stomachache (17), stomach discomfort (2), hunger-related stomach cramps (1), flatulence (1)	Although you had an upset stomach (e.g., stomachache, flatulence), you still persevered in going to work
9	General malaise	17	sore and swollen (10), exhausted (1), whole body ache (1), panidrosis (1), malaise (1), discomfort from sitting or standing for too long (1)	Although you felt whole body fatigue or discomfort, you still persevered in going to work
10	Nausea or emesis	14	Nausea (9), emesis (5)	Although you experienced nausea and felt like vomiting, you still persevered in going to work
11	Eye discomfort	11	Ophthalmodynia (7), asthenopia (1), dry eyes (1), giddiness (1), blurring of vision (1)	Although you had other physical symptoms, you still persevered in going to work
12	Pain in the neck or cervical spine	10	Cervical pain (6); neck pain (4)	
13	Diarrhoea	9	Diarrhoea (9)	
14	Lack of sleep	7	Lack of sleep (5), insomnia (2)	
15	Pregnant reaction	4	Pregnant reaction (2), pregnancy discomfort (1), abdominal pain during pregnancy (1)	
16	Toothache	2	Toothache (2)	
17	Interpulmonary neuralgia	1	Interpulmonary neuralgia (1)	
18	nosebleed	1	Nosebleed (1)	

### Study 2

The aim of Study 2 was to test the factor structure of the NPQ generated in study 1 through exploratory factor analysis (EFA) and confirmatory factor analysis (CFA). The reliability and validity of the questionnaire were also tested using reliability analysis and criterion validity analysis to obtain the formal version of the NPQ.

#### Participants and Procedure

In this study, the formal NPQ survey was conducted through convenience sampling to test its applicability. A total of 650 hardcopy questionnaires were distributed to four hospitals in Henan province, China, and 572 valid questionnaires were collected, with an effective response rate of 87.69%. They ages ranged from 19 to 53 years, with an average age of 30.61 (SD = 5.21) years. Nursing tenure ranged from 0.5 to 30 years, with an average of 8.72 years (SD = 5.56). The departments in which nurses worked included internal medicine, surgery, ophthalmology, paediatrics, obstetrics and gynaecology, emergency room, and outpatient service. Other demographic variables were shown in Table 1, which included gender, marital status, and technical title.

Due to the study data being cross-sectional, half of the total data was randomly selected for EFA and for CFA. The Excel software function of RANDBETWEEN was adopted to generate a list of random assignment values that consisted of 0 and 1, which represented the part of data that would be used for EFA or CAF in the next stage, respectively. We obtained 288 items for EFA and 284 items for CFA. After importing the data into the analysis software, the corresponding statistical tests were conducted. Finally, the formal version of the NPQ was formed.

During the study, four trained researchers first contacted the heads of nursing departments in these hospitals to explain the purpose of the study and related matters of the NPQ. Participants completed the NPQ voluntarily and anonymously. Then the questionnaires were uniformly collected at the designated location and finally summarised by the researchers. The Ethical Review Board of the Institution of Psychology and Behaviour, Henan University, approved the design of this study. All participants provided oral informed consent prior to completing the NPQ, and the confidentiality principle of the questionnaire was explained in the instructions.

#### Measures

General demographic characteristics such as gender, age, tenure, marital status, technical title, and work units were collected.

##### Nurse presenteeism

In the item generation stage, 11 items were selected to constitute the NPQ. An example of the items is: “Although you felt dizzy or had a headache, you still persevered in going to work.” The participants were required not repeat reports (just select 0) if the following situation occurs in the same sick attendance behaviour. The NPQ adopted a four-point Likert scale rating system ranging from 0 to 3 for these 11 items (0 = never, 1 = once, 2 = 2–5 times, 3 = more than five times), with high scores representing more frequent instances of presenteeism. In this study, the internal reliability coefficient of the NPQ was 0.94.

##### Sickness presenteeism

Sickness Presenteeism Questionnaire was adopted among nurses (Lu et al., 2013a) as one of the criterion-related variables, which were introduced in Study 1. In this study, the internal reliability coefficient of SPQ was 0.89.

##### Emotional exhaustion

Emotional exhaustion, a dimension of job burnout, was assessed using the Emotional Exhaustion Scale (EES) of Chinese Maslach Burnout Inventory (CMBI), which had previously been determined to have adequate reliability and validity in a Chinese sample (Li and Wu, 2005; Li et al., 2005). It was assessed by five items, such as: “I feel burned out from my work.” The items were rated using a seven-point Likert scale, ranging from 1 (completely inconsistent) to 7 (completely consistent), with higher scores representing greater emotional exhaustion. In this study, the internal reliability coefficient of EES was 0.90.

##### Health-related productivity loss

Health-related productivity loss was assessed using the Chinese version of the Stanford Presenteeism Scale (SPS-6) (Zhao et al., 2010), which has been widely used to assess the impact of health problems on an individual’s productivity (Koopman et al., 2002). It contains six items, including two dimensions of work constraints (with four items, e.g., “Despite having my health problem, my work pressure is more difficult to adjust”) and avoiding distraction (with two items, e.g., “Despite having my health problems, I was able to concentrate and finish the work,” requiring reverse scoring). All the items were scored on a five-point Likert scale, ranging from 1 (completely disagree) to 5 (totally agree). Higher SPS-6 scores reflected greater loss of health-related productivity caused by presenteeism of the participants. In this study, the internal reliability coefficient of the SPS-6 was 0.86.

##### General health

General health was assessed using the 12-item General Health Questionnaire (GHQ-12), which required nurses to report their perceptions regarding their health conditions (Goldberg et al., 1997). The questionnaire focuses on the two areas of normal dysfunction and recently appearing distressing situations to assess an individual’s current state. It seeks to identify any differences from the usual state (Fryers et al., 2004). It contains six positive items (e.g., “Have you been able to enjoy daily activities?,” requiring reverse scoring) and six negative items (e.g., “Have you felt unhappy or depressed?”). All the items are scored on a four-point Likert scale, ranging from 1 (never) to 4 (usually). Higher scores reflect a lower health level (Gnambs and Staufenbiel, 2018). The questionnaire has been successfully conducted in Chinese samples and has already been proven to have good psychometric properties (Li and Li, 2015). In this study, the internal reliability coefficient of the GHQ-12 was 0.81.

#### Statistical Analysis

SPSS 22.0 and Amos 22.0 were used to analyse data. First, the independent-samples *t* test was used to evaluate the discrimination of items, and the correlation analysis was used to calculate the correlation between the item and complete questionnaire. Then, the EFA was used to determine the factor structure and the loading of items. Next, the CFA was adopted to further determine the NPQ structure. Finally, criterion-relative correlation analysis was conducted to access the criterion-relative validity of the NPQ.

## Results

### Item Analysis

To test the discrimination of items, we calculated each participant’s total o NPQ score. Then participants were divided into two groups based on their scores, from high to low. Specifically, the participants who scored the top 27% of participants with the highest scores constituted the high-score group while the bottom 27% of participants with the lowest scores constituted the low-score group Then, the differences in each item between the two groups were analysed. The results showed that all items had significant differences between the two groups (*t* = 22.67∼36.11, *p* < 0.01). Subsequently, the correlation between each item and the total score was analysed to test the homogeneity of those items. The results showed that the correlation coefficients between each item and the total score were significant (*r* = 0.74∼0.85, *p* < 0.01).

### EFA

An EFA was conducted on the 11 items to determine the factor structure of the NPQ. The Kaiser-Meyer-Olkin (KMO) value of sampling adequacy was 0.95, and the result of Bartlett’s test for sphericity showed a significant difference [χ^2^/df = 2197.35 (55), *p* < 0.001], which demonstrated that the sample was appropriate for factor analysis (Tabachnick and Fidell, 2013). Meanwhile, the maximum likelihood method was adopted, the factor was extracted based on the eigenvalue greater than 1, and the optimal skew method was used to rotate the factor. The results showed that only one factor was extracted, with a cumulative variance contribution rate of 63.06%. Besides, the internal reliability coefficient of the NPQ was 0.94. The factor loading results are shown in Table 5.

**TABLE 5:** Factor loading of the NPQ.

		EFA	CFA
		
Items		Load	Load
1	Although you had a fever, you still persevered in going to work	0.71	0.71
2	Although you felt dizzy or had a headache, you still persevered in going to work	0.80	0.77
3	Although you felt abdominal pain (including menstrual pain), you still persevered in going to work	0.71	0.67
4	Although you had a cold (e.g., stuffy nose or cough), you still persevered in going to work	0.75	0.71
5	Although you felt chest distress, shortness of breath, or palpitations, you still persevere in going to work	0.76	0.75
6	Although you felt discomfort in the lower back, you still persevered in going to work	0.72	0.70
7	Although you felt pain or swelling in limbs (and joints), you still persevered in going to work	0.78	0.73
8	Although you had an upset stomach (e.g., stomachache, flatulence), you still persevered in going to work	0.81	0.79
9	Although you felt whole body fatigue or discomfort, you still persevered in going to work	0.86	0.82
10	Although you experienced nausea and felt like vomiting, you still persevered in going to work	0.76	0.77
11	Although you had other physical symptoms, you still persevered in going to work	0.82	0.79

*The number of items is the same as in Table 3. The CFA loads are the standardized estimates.*

### CFA

To verify the validity of the NPQ, AMOS 22.0 was used for CFA. When all 11 items were loaded into a single factor, the result generally showed a good fit (χ^2^ = 143.92, df = 41, χ^2^/df = 3.51, *p* = 0.00, comparative fit index [CFI] = 0.95, goodness of fit index [GFI] = 0.92, adjusted goodness of fit index [AGFI] = 0.87, root mean square error of approximation [RMSEA] = 0.09, standardised root mean square residual [SRMR] = 0.04). Meanwhile, the internal reliability coefficient of this questionnaire was 0.93.

### Correlation Analysis

To further verify the validity of the NPQ, a correlation analysis between criterion-relative variables and nurse presenteeism was conducted, as shown in Table 6. The results revealed a positive and highly significant correlation between the NPQ and SPQ scores (*r* = 0.84, *p* < 0.01). The NPQ was moderately positively correlated with SPS-6 (*r* = 0.24, *p* < 0.01), GHQ-12 (*r* = 0.33, *p* < 0.01), and EES (*r* = 0.45, *p* < 0.01). These results proved the beneficial criterion-relative validity of the NPQ.

**TABLE 6:** Correlate analysis between NPQ and its criterion-related variables (*r*, *n* = 284).

		***M***	***SD***	**NPQ**	**SPQ**	**SPS-6**	**GHQ-12**	**EES**
1	NPQ	1.50	0.84	(0.93)				
2	SPQ	1.61	1.05	0.84**	(0.88)			
3	SPS-6	2.58	0.69	0.24**	0.21**	(0.85)		
4	GHQ-12	2.04	0.50	0.33**	0.30**	0.37**	(0.83)	
5	EES	3.56	1.37	0.45**	0.43**	0.49**	0.66**	(0.91)

*NPQ: Nurse Presenteeism Questionnaire; SPQ: Sickness Presenteeism Questionnaire; SPS-6: the Chinese version of the Stanford Presenteeism Scale; GHQ-12: the 12-item General Health Questionnaire; EES: emotional exhaustion scale of CMBI.*

The Cronbach’s α values are shown in brackets.

**p < 0.01.

## Discussion

This study aimed to develop a multi-item presenteeism questionnaire for Chinese nursing occupations in order to explore the relationship between presenteeism and its related variables in depth and provide an alternative and effective measurement tool for future research. Therefore, based on the previous single-item and two-item questionnaire for presenteeism, the core disease symptoms of nurse presenteeism were investigated and incorporated into the NPQ after taking into consideration the existing literature and the compressive feedback provided by participants.

After being succinctly summarised based on the information collected from the open-ended questionnaire, 10 core symptoms of nurse presenteeism were obtained. The frequency of these symptoms reached 90.02% and an item “other symptoms” was added as a supplement, which indicated the wide coverage of these items. The results of item analysis revealed that there was a significant high correlation (correlation coefficient between 0.74 and 0.85) between each item and the total NPQ score. Meanwhile, the overall internal consistency reliability coefficient was higher than 0.9, which revealed the NPQ’s good reliability. The EFA results indicated that all 11 items loaded in a single factor with a high factor load and that contributed to 63.06% of the total variance, which supported the single dimensional structure of NPQ. The subsequent results of CFA also confirmed the validity of the NPQ. The above preliminarily results proved the good reliability and validity of the NPQ.

Furthermore, some related variables of presenteeism were selected to verify the external validity of the NPQ. First, based on the content of this questionnaire, the SPQ that was developed by Lu et al. (2013a) and the NPQ in our study could both be used to measure nurse presenteeism; thus, they should be highly positively correlated, which is consistent with our results. In addition, compared with the SPQ, the internal reliability coefficient of the NPQ in our study reached 0.93 and above, which is higher than the internal reliability coefficient of the SPQ in previous studies on nursing participants (e.g., Zhang et al., 2018; Li et al., 2019). These results supported the good reliability of the NPQ. Second, presenteeism is inseparable from personal health. According to the recovery theory (Meijman and Mulder, 1998), people need enough resources to recover their physical and mental energy after work. If the recovery is insufficient and the energy continues to be consumed, people will be drained of energy to cope with their present job demand, further leading to long-term damage to their health. Empirical studies have also indicated that presenteeism, as a vital predictor of self-rated health status (e.g., Dellve et al., 2011; Gustafsson and Marklund, 2011), could lead to negative impacts on individuals’ physical and mental health (e.g., Lu et al., 2013a; Conway et al., 2014) and cause a large amount of productivity loss for individuals and their organisations (e.g., Robertson and Cooper, 2011; Li et al., 2019). These studies support the finding in our study that presenteeism is negatively correlated with general health and health-related productivity loss. Finally, the positive correlation of presenteeism and emotional exhaustion in our study was also consistent with previous research. Demerouti et al. (2009) found a reciprocal relationship between presenteeism and emotional exhaustion in their longitudinal research, which pointed out that emotional exhaustion at the baseline (T1), led to presenteeism at 12 months (T2), which in turn resulted in more emotional exhaustion at 6 months (T3). It was explained that, when individuals felt exhausted, they would invest greater efforts to avoid the negative effects of progressive energy depletion, further leading to presenteeism, which in turn resulted in enhanced feelings of exhaustion. In addition, studies by Dellve et al. (2011) and Lu et al. (2013b) verified the positive relationship between presenteeism and emotional exhaustion, considering Chinese employees and Swedish healthcare workers as samples, respectively. Therefore, all these results confirmed the good criterion-related validity of the NPQ.

## Limitations and Future Research

In this study, a multi-item NPQ was developed and its good reliability and validity were confirmed. Thus, it is an effective measurement tool for future in-depth research of presenteeism and offers a degree of convenience in exploring the complex mechanisms between presenteeism and its related variables. However, some limitations of this research should be considered. First, the questionnaire was developed with Chinese nurses as samples, and the item pool was formed based on their responses. Hence, although the content of this questionnaire reflected the professional characteristics of nurses, its applicability to other occupational samples is uncertain and will necessitate further studies. In addition, although our questionnaire focused on the physical symptoms of female nurses, there were still a few male participants in our study 2. However, in China, male nurses account for about 2% of total number of registered nurses (Sun and Zhao, 2019). In present study, the proportion of male nurses was consistent with the prior study. Therefore, we did not remove the male samples in study 2. Future studies should recruit more male samples and conduct the gender invariance test to examine whether the questionnaire in present study could be applied in male nurses. Second, in the process of the items pool generation of the NPQ, core symptoms constituting the top 90% of responses were selected as the main items. Although “other symptom” was added as a supplement item, some symptoms were discarded, which may have affected the effectiveness of the questionnaire to a certain extent. Third, the Covid-19 pandemic is not completed overcome in a global context, and pandemic may influence our results in present study. However, on the current conditions of China, the work and life of residents has completely recovered. In addition, before study 2 was carried out, the cooperative hospitals had confirmed the nurses in the region had returned to normal work for more than 6 months. Furthermore, according to the Law of the People’s Republic of China on Prevention and Treatment of Infectious Diseases, when nurses are infected with infectious diseases, for the sake of the health of themselves and others, they should be quarantined for treatment. Therefore, nurses have no chance to persist with work who have infectious diseases like SARS and Covid-19.

## Data Availability Statement

The raw data supporting the conclusions of this article will be made available by the authors, without undue reservation.

## Ethics Statement

The studies involving human participants were reviewed and approved by Henan University Institutional Review Board. The patients/participants provided their written informed consent to participate in this study.

## Author Contributions

YL was the principal investigator who generated the idea and designed the study. GS, SW, and KF were the primary writers of the manuscript and approved all changes. GS and WW assisted with the data input and data analysis. SG assisted with the data collection. All authors were involved in developing, editing, reviewing, and providing feedback for this manuscript and have given approval of the final version to be published.

## Conflict of Interest

The authors declare that the research was conducted in the absence of any commercial or financial relationships that could be construed as a potential conflict of interest.
